# Correlation between spinopelvic sagittal balance and vertebral fractures in postmenopausal women

**DOI:** 10.1590/0100-3984.2025.0037-en

**Published:** 2025-10-21

**Authors:** Leonor Garbin Savarese, Otávio Takassi Moritsugu, Luciana Mendes Cangussu Oliveira, Daniela Cristina Carvalho de Abreu, Francisco José Albuquerque de Paula, Marcello Henrique Nogueira-Barbosa

**Affiliations:** 1 Ribeirão Preto Medical School, University of São Paulo (USP), Ribeirão Preto, SP, Brazil

**Keywords:** Spine, Radiography, Spinal fractures, Osteoporosis., Coluna vertebral, Radiografia, Fraturas da coluna vertebral, Osteoporose.

## Abstract

**Objective:**

To investigate the relationship between spinopelvic alignment and vertebral fracture in postmenopausal women with osteoporosis.

**Materials and Methods:**

This was a retrospective cross-sectional study including 93 women diagnosed with osteopenia or osteoporosis by densitometry between June 2017 and March 2018. Using the software Surgimap to analyze lateral X-rays of the spine and pelvis, we measured the following spinopelvic parameters: pelvic incidence (PI), pelvic tilt (PT), sacral slope (SS), sagittal vertical axis (SVA), global tilt (GT), spinosacral angle (SSA), T1 pelvic angle (TPA), lumbar lordosis (LL), and thoracic kyphosis (TK). The spinopelvic parameters were assessed in relation to fracture occurrence by estimating prevalence ratios. Two groups (patients with and without fractures) were compared on the basis of their spinopelvic parameters. Vertebral fractures were graded by the Genant classification, and the spinal deformity index (SDI) was calculated as the sum of the grades. The SDI was found to correlate with spinopelvic parameters. Intraobserver and interobserver reliability for the measurement of the spinopelvic parameters was evaluated.

**Results:**

The GT correlated significantly with the presence of fractures; the incidence of fracture was found to increase by 2.1% for every 1-degree increase in the GT. The presence of fractures was not found to correlate significantly with the SS, PT, PI, LL, TK, SVA, or SSA. The GT was significantly greater in the group with fractures than in the group without fractures. The SDI correlated significantly with global sagittal balance, as measured by the GT.

**Conclusion:**

Fractures seem to be more prevalent among women with a higher GT. The SDI appears to correlate well with global sagittal balance, as assessed by the GT.

## Abstract (pt)

**Objetivo:**

Investigar a relação entre o alinhamento espinopélvico e a presença de fraturas vertebrais em mulheres com osteopenia na pós-menopausa.

**Materiais e Métodos:**

Entre junho de 2017 e março de 2018, 93 pacientes do sexo feminino que receberam diagnóstico de osteopenia ou osteoporose no exame de densitometria foram incluídas neste estudo transversal retrospectivo. Os parâmetros espinopélvicos incidência pélvica (IP), versão pélvica (VP), inclinação sacral (IS), eixo vertical sagital (EVS), versão global (VG), ângulo espinossacral (ASS), ângulo T1 pélvico (ATP), lordose lombar (LL) e cifose torácica (CT) foram mensurados nas radiografias panorâmicas da coluna e pelve com a paciente na posição ortostática utilizando o *software* Surgimap. Os parâmetros espinopélvicos foram relacionados com a ocorrência de fratura estimando-se as razões de prevalência. Foi realizada comparação dos grupos (presença e ausência de fratura) quanto aos parâmetros espinopélvicos. As fraturas vertebrais foram graduadas segundo a classificação de Genant, e o índice de deformidade espinhal (IDE) foi calculado como a soma dos graus. O IDE foi relacionado com os parâmetros espinopélvicos. A reprodutibilidade da mensuração dos parâmetros espinopélvicos foi avaliada.

**Resultados:**

Foi observada correlação significativa entre os valores da VG e a presença de fraturas, em que a cada aumento de 1 grau na VG a prevalência de fratura vertebral aumentou, em média, 2,1%. Não foi encontrada correlação entre os parâmetros espinopélvicos IS, VP, IP, LL, CT, EVS e ASS e a presença de fraturas. A comparação dos grupos com e sem fratura demonstrou diferença estatisticamente significante em relação ao parâmetro VG, que foi mais alto no grupo com fratura. O IDE se correlacionou significativamente com o alinhamento sagital global, mensurado pela VG.

**Conclusão:**

Mulheres com maior VG apresentaram maior prevalência de fraturas. O IDE se correlacionou significativamente com o alinhamento sagital global, mensurado pela VG.

## INTRODUCTION

Osteoporosis is a significant public health problem characterized by reduced bone mass and density, resulting in skeletal fragility and an increased risk of fractures, especially in areas such as the hip, wrist, and spine^**(^1^)**^. Vertebral fractures are often the first manifestation of bone fragility and are associated with spinal deformities, chronic low back pain, and a significant decline in health-related quality of life^**(^2^)**^.

Given the physical, psychosocial, and public health impact of vertebral fractures, it is essential to identify patients with osteoporosis who are at high risk of developing them^**(^3^)**^. The occurrence of a vertebral fracture significantly increases the likelihood of future fractures, with women over 50 years of age representing the group at highest risk^**(^4^)**^. Although the assessment of vertebral fracture risk has historically been based on bone mineral density (BMD), evidence suggests that BMD alone is insufficient to fully predict the risk of such fractures^**(^5^)**^.

In recent years, the relationship between sagittal spinal alignment and fragility fractures has received increasing attention^**(^6^-^8^)**^. Studies have shown that patients with osteoporosis are more likely to show sagittal misalignment than are individuals without the disease^**(^9^)**^. In addition, the presence of vertebral fractures has been shown to alter the sagittal balance of the spine, playing an important role in the development of new fractures^**(^10^)**^. With aging, kyphosis of the thoracic spine tends to increase, causing an anterior tilt of the trunk^**(^11^)**^. The pelvis often compensates for this posture through retroversion, but patients unable to perform this compensation experience imbalances that, in addition to increasing the risk of falls, contribute to the development of new osteoporotic fractures^**(^12^)**^. In patients with osteoporosis, sagittal spinal misalignment has been identified as an independent risk factor for subsequent vertebral fractures^**(^13^)**^. Nevertheless, studies focusing on the influence that vertebral fractures have on global sagittal balance, especially studies involving postmenopausal women, are still scarce.

We hypothesized that changes in sagittal alignment would serve as a relevant prognostic indicator of the risk of vertebral fractures in patients with osteopenia or osteoporosis. Therefore, the aim of this study was to investigate the relationship between sagittal spinal alignment and the presence of vertebral fractures in postmenopausal women.

## MATERIALS AND METHODS

### Study population

Patients who underwent bone densitometry between June 2017 and March 2018 were included in this retrospective cross-sectional study. The study was approved by the local research ethics committee, and the requirement for informed consent was waived. The inclusion criteria were as follows: being a woman; being over 50 years of age; having a lumbar spine BMD T-score < -1.0 SD; and being able to walk, sit, and stand without an assistive device. Patients with severe cardiovascular or pulmonary disease were excluded, as were those with renal or hepatic insufficiency, those with uncontrolled type 2 diabetes mellitus, those with bone loss secondary to other diseases (rheumatoid arthritis, osteomalacia, or osteogenesis imperfecta), those with neurological disorders (Parkinson’s or Alzheimer’s disease), those with a history of spine or hip surgery, and those in whom the radiographic technique was deemed inappropriate. A total of 153 patients were considered eligible. Of those, 60 were excluded: 32 because of secondary bone loss (due to rheumatoid arthritis in seven, osteogenesis imperfecta in 21, and other diseases in four); 10 due to severe cardiovascular disease; 12 because of neurological disorders (Parkinson’s in five and Alzheimer’s in seven); four because of a history of hip surgery; and two because the radiographic technique had been inappropriate, which made it impossible to calculate the spinopelvic parameters. Therefore, the final sample comprised 93 female patients.

### BMD measurement

To measure BMD, dual energy X-ray absorptiometry scans of the lumbar spine and femoral neck were obtained. All of the scans were acquired in the same scanner (Discovery CI/WI, 4500W/CE; Hologic, Bedford, MA, USA). A T-score between -1.0 SD and -2.5 SD is indicative of osteopenia, whereas a T-score ≤ -2.5 SD is indicative of osteoporosis^**(^8^)**^. According to the National Osteoporosis Foundation^**(^8^)**^, severe or established osteoporosis is characterized by a T-score < -2.5 SD, together with at least one fragility fracture.

### Radiographic evaluation

The radiographic evaluation of the spine had two different objectives: to identify any vertebral fractures; and to evaluate sagittal balance by measuring spinopelvic parameters. For each patient, a panoramic X-ray was obtained, in a lateral view, with a computed radiography system (Kodak CR Long Length Vertical Imaging System; Carestream Health, Rochester, NY, USA). The patients were imaged while standing, with their arms on a support, shoulders at 30° of flexion, and elbows slightly flexed, as described in the literature^**(^14^)**^, in order to minimize any postural compensations.

**Figure 1:**
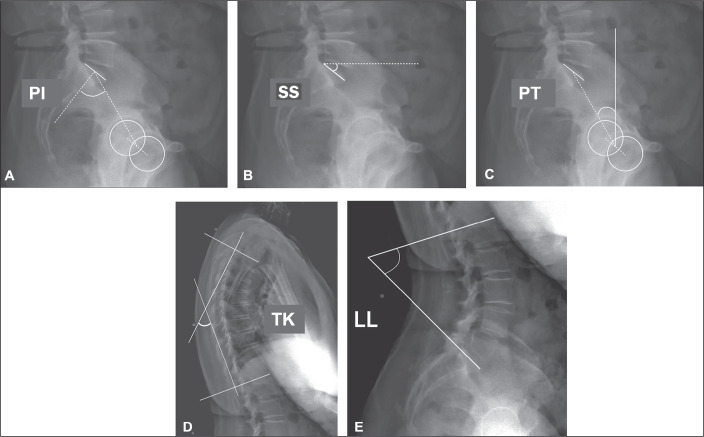
Measurement of the spinopelvic parameters PI, SS, PT, TK, and LL.

**Figure 2:**
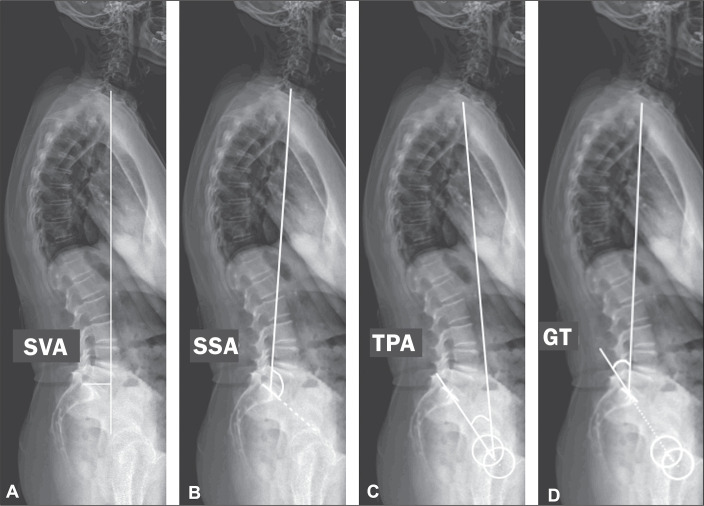
Measurement of the spinopelvic parameters SVA, SSA, TPA, and GT.

To measure the spinopelvic parameters and vertebral curvature angles, we used Surgimap software (Nemaris Inc., New York, NY, USA). The following parameters were evaluated (Figures 1 and 2): sacral slope (SS); pelvic tilt (PT); pelvic incidence (PI); lumbar lordosis (LL); thoracic kyphosis (TK); sagittal vertical axis (SVA); spinosacral angle (SSA); T1 pelvic angle (TPA); and global tilt (GT). The SS corresponds to the angle formed between the upper endplate of S1 and a horizontal line. The PT corresponds to the angle formed between a vertical line originating at the center of the femoral head and a line running from the center of the femoral head to the midpoint of the S1 endplate. The PI corresponds to the angle formed by a line running perpendicular to the sacral plateau and a line connecting its midpoint with the center of femoral rotation. The degree of LL is determined by measuring the Cobb angle from the superior endplate of S1 to the superior endplate of L1. The degree of TK is determined by measuring the Cobb angle from the inferior endplate of T12 to the superior endplate of T1. The SVA is the measurement of the horizontal distance between the plumb line of C7 and the vertical line passing through the posterosuperior point of S1. The SSA corresponds to the angle formed between the line passing from the center of C7 to the center of the endplate of S1 and the surface of the sacral endplate. The TPA corresponds to the angle formed by a line running from the geometric center of the femoral heads to the center of the T1 vertebral body and a line running from the geometric center of the femoral heads to the center of the superior endplate of S1. The GT is defined as the angle formed by a line running from the center of the superior sacral endplate to the center of the C7 vertebral body and a line running from the geometric center of the femoral heads to the center of the sacral endplate^**(^15^)**^. The contours of the femoral heads were marked, and lines were drawn adjacent to the superior plateau of S1, superior plateau of L1, inferior plateau of T12, superior plateau of T1, and inferior plateau of C2. From those markings, the software automatically calculated the spinopelvic parameters and vertebral curvatures.

Radiographic images of the thoracic spine and lumbar spine were used in order to assess the presence of vertebral fractures and to classify the severity of any such fractures. The standard employed to evaluate vertebral fractures was the semiquantitative grading system using anteroposterior and lateral X-rays, developed by Genant et al. in 1993^**(^16^)**^. According to this grading system, a T4-L4 vertebral deformity with a reduction in vertebral height of more than 20% is defined as a fracture. There are four Genant grades, which are differentiated as follows^**(^10^)**^: grade 0 = no fracture; grade 1 = mild fracture, defined as a 20-25% reduction in vertebral height (in comparison with normal adjacent vertebrae); grade 2 = moderate fracture, defined as a 25-40% reduction in vertebral height; and grade 3 = severe fracture, defined as a > 40% reduction in vertebral height. Patients were divided into two groups: those with at least one vertebral fracture and those without any such fractures. The spinal deformity index (SDI) was calculated as the sum of the grades from T4 to L4^**(^17^)**^.

### Image evaluation

The first examiner (observer 1), a radiologist with six years of experience, was responsible for measuring the spinopelvic parameters in all 93 of the patients in the sample. Repeated measurements were performed on the same cases, with a one-month interval between the first and second measurements to allow intraobserver reliability to be evaluated. The second examiner (observer 2), a radiologist with 10 years of experience, was blinded to the results of the first examiner and performed the same measurements on images of 47 of the 93 patients, to allow interobserver reliability to be evaluated. The more experienced radiologist (observer 2) assessed the presence of fractures.

### Statistical analysis

Intraobserver and interobserver reliability for the measurement of spinopelvic parameters was analyzed by calculating the intraclass correlation coefficient (ICC), with a 95% confidence interval. The ICC values (i.e., levels of reliability) were classified as follows: < 0.50 = poor; 0.50-0.75 = moderate; 0.75-0.90 = good; and > 0.90 = excellent. A simple log-binomial regression model was used in order to relate the parameters of interest (SS, PT, PI, LL, TK, SVA, SSA, TPA, and GT) with the occurrence of fractures, by estimating prevalence ratios. The Mann-Whitney test was used in order to compare the two groups (with and without fractures) regarding the spinopelvic parameters. Spearman’s correlation coefficient (*r*) was employed to relate the SDI to the spinopelvic parameters. The reliability analysis was performed by using R software, version 4.1.0 (The R Project for Statistical Computing, Vienna, Austria). The remaining analyses were performed with the Statistical Analysis System, version 9.4 (SAS Institute Inc., Cary, NC, USA). A significance level of 5% was adopted. The statistical power (probability of a type II error, 1 - β) of our sample of 93 participants was 0.86, with a probability of a type I error (α) of 5%.

## RESULTS

Among the 93 women in the study sample, the mean age was 67.5 ± 9.4 years (range, 51-82 years). The sagittal parameters of the groups with and without fractures are summarized in Table 1. The groups with and without fractures differed significantly in terms of the TPA (*p* = 0.05) and GT (*p* = 0.03), although not in terms of the SS, PT, PI, LL, TK, SVA, or SSA. At least one vertebral fracture was identified in 37 patients (39.8%), whereas the remaining 56 patients (60.2%) had no discernible fractures. Of a total of 99 fractures identified, 37 were located below L1 and 62 were located above T12.

**Table 1:** Comparison between patients without fracture (n = 56) and those with fracture (n = 37), in terms of spinopelvic parameters.

Parameter	Patients without fracture Mean ± SD	Patients with fracture Mean ± SD	*P*
SS(°)	33.3 ± 8.7	31.9 ± 11.7	0.31
PT (°)	18.2 ± 10.2	20.1 ± 7.3	0.19
PI (°)	51.4 ± 13.8	51.4 ± 13.9	0.95
LL(°)	53.4 ± 12.2	51.6 ± 16.7	0.72
TK(°)	38.2 ± 12.0	41.9 ± 15.8	0.21
SVA (mm)	30.8 ± 19.1	40.2 ± 35.1	0.51
SSA(°)	122.2 ± 8.1	118.9 ± 14.4	0.08
TPA(°)	14.0 ± 7.8	18.4 ± 13.1	0.05
GT (°)	19.0 ± 10.2	23.3 ± 10.5	0.03
SDI	-	1.24 ± 2.15	-

Table 2 presents the correlations between the spinopelvic parameters and the presence of vertebral fracture. A statistically significant relationship was observed between the GT and the presence of fracture; for each 1-degree increase in GT, the prevalence of fracture increased, on average, by 2.1%. Table 3 shows the correlations between the spinopelvic parameters and the SDI. A statistically significant correlation was observed between the SDI and the GT (*p* < 0.01). Table 4 presents the intraobserver and interobserver reliability for the spinopelvic parameters. The level of intraobserver reliability was moderate (ICC = 0.71), whereas the level of interobserver reliability was good (ICC = 0.79).

**Table 2:** Relationship between the spinopelvic parameters of interest and the occurrence of fracture.

Parameter	PR (95% Cl)	*P*
SS(°)	0.99 (0.96-1.02)	0.44
PT (°)	1.01 (0.99-1.03)	0.39
PI (°)	1.00 (0.98-1.02)	0.99
LL(°)	0.99 (0.97-1.01)	0.47
TK(°)	1.01 (0.99-1.03)	0.16
SVA (mm)	1.01 (0.99-1.02)	0.26
SSA(°)	0.97 (0.95-1.00)	0.10
TPA(°)	1.01 (0.99-1.,03)	0.29
GT (°)	1.02 (1.00-1.04)	0.04

PR, prevalence ratio (estimated by log-binomial regression); 95% CI, 95% confidence interval.

**Table 3:** Correlation between the spinopelvic parameters of interest and the SDI.

Parameter	Spearman’s correlation	*P*
SS(°)	-0.12	0.26
PT (°)	0.20	0.05
PI (°)	0.02	0.83
LL(°)	-0.02	0.81
TK(°)	0.17	0.11
SVA (mm)	0.07	0.51
SSA(°)	-0.20	0.05
TPA(°)	0.19	0.06
GT (°)	0.28	< 0.01

**Table 4:** Intraobserver and interobserver reliability for the measurement of the spinopelvic parameters of interest.

Parameter	Reliability	
	Intraobserver ICC (95% Cl)	Interobserver ICC (95% Cl)
SS(°)	0.89 (0.84-0.93)	0.79 (0.66-0.88)
PT (°)	0.86 (0.79-0.90)	0.93 (0.87-0.96)
PI (°)	0.82 (0.74-0.88)	0.85 (0.75-0.92)
LL (°)	0.92 (0.88-0.95)	0.90 (0.83-0.94)
TK(°)	0.93 (0.90-0.96)	0.97 (0.95-0.98)
SVA (mm)	0.98 (0.97-0.98)	0.98 (0.97-0.99)
SSA(°)	0.89 (0.85-0.93)	0.84 (0.74-0.91)
TPA(°)	0.71 (0.59-0.80)	0.89 (0.82-0.94)
GT (°)	0.88 (0.82-0.92)	0.93 (0.88-0.96)

95% CI, 95% confidence interval.

## DISCUSSION

To our knowledge, this is the first study to demonstrate a correlation between global sagittal alignment, as assessed by calculating the GT, and the occurrence of fractures. As previously stated, we found that the prevalence of fractures increased by an average of 2.1% for every 1-degree increase in GT, as well as that the spinopelvic parameters SS, PT, PI, LL, TK, SVA, and SSA did not correlate with the presence of fractures. The comparison between the groups with and without fractures showed a statistically significant difference in the GT, which was greater in the former group. The SDI also correlated significantly with the GT.

Previous studies have demonstrated that patients with osteoporosis have worse sagittal alignment than do individuals without the disease^**(^9^,^18^,^19^)**^ and that individuals with vertebral compression fractures have worse sagittal alignment than do age-matched individuals without such fractures^**(^20^)**^. Multiple fractures are known to contribute to sagittal malalignment in patients with osteoporosis^**(^6^,^10^)**^. However, sagittal malalignment of the spine has been consistently reported as an independent risk factor for subsequent vertebral fractures in individuals with osteoporosis^**(^13^,^21^-^23^)**^.

Other studies have found that PT and SVA values are higher in individuals with vertebral fractures than in controls^**(^20^,^22^,^24^)**^. Matsunaga et al.^**(^18^)**^ found PT and SVA values to be higher in patients with at least two vertebral fractures than in individuals without any such fractures. Dai et al.^**(^13^)**^ conducted a study of 1,044 postmenopausal women with osteoporosis and observed that LL, SS, and PI values were lower in those who developed fractures, postulating that this specific pattern would be a predictor of risk for the development of fractures. In keeping with our findings, those authors found no differences between the women who did and did not develop fractures during follow-up, in terms of the TK, PT, or SVA. The fact that we did not identify a correlation between SVA and the presence of fractures supports the idea that, when the sagittal plane is being evaluated, the SVA should not be analyzed in isolation. A recent study demonstrated that intervertebral disc signal abnormalities on magnetic resonance imaging correlate well with the GT and TPA but not with the SVA^**(^25^)**^, highlighting the potential importance of using these angles in postural assessments. Analysis of the GT is advantageous for assessing global alignment because it takes into account pelvic retroversion and trunk anteversion and is not influenced by postural or radiographic calibrations^**(^26^)**^. In addition, the GT, SVA, and PT correlated strongly with quality of life^**(^27^)**^. In our study sample, the GT parameter was significantly greater in the patients with fractures, suggesting an anterior shift in sagittal balance associated with pelvic retroversion.

Although we observed lumbar lordosis to be, on average, 1.8-degree less in the fracture group, the difference was not statistically significant. In a longitudinal study, Yokoyama et al.^**(^28^)**^ demonstrated that a fracture at the lower lumbar level is associated with greater anterior displacement of the upper vertebrae, which requires more significant compensatory changes to maintain sagittal balance. In patients with fractures in the lower lumbar spine, LL decreased significantly, so that the thoracic spine was unable to compensate to restore sagittal balance, despite the reduction in kyphosis. In contrast, in patients with fractures in the thoracic spine or at the thoracolumbar junction, the deterioration in sagittal balance was mild, even in cases of severe vertebral collapse^**(^28^)**^. In our study, most fractures occurred above T12, which could explain the fact that neither the SVA nor the PT were found to correlate with the presence of fractures.

Hu et al.^**(^29^)**^ investigated the impact of vertebral compression fractures on global sagittal alignment in elderly patients with osteoporosis. They found that the patients with vertebral compression fractures had worse global sagittal alignment, with a greater TPA and global sagittal angle, in comparison with those without such fractures. Those results are consistent with the findings of the present study. However, to our knowledge, ours is the first study to use the GT parameter to compare groups with and without fractures, thus complementing the existing data in the literature. In the Hu et al. study^**(^29^)**^, the number and severity of vertebral compression fractures correlated positively with unfavorable global sagittal alignment. Our findings are consistent with those of that study, given that we found the GT to correlate with fracture severity, as measured by the SDI.

Our study has some limitations. The study sample consisted only of individuals with osteopenia and osteoporosis; we did not evaluate individuals with normal BMD, which could have enabled further comparisons regarding fracture incidence. In addition, because this was a cross-sectional study, we evaluated associations only between parameters, and it was therefore not possible to infer any cause-and-effect relationships. There is a need for longitudinal studies to demonstrate changes after vertebral body collapse. Another relevant limitation is that compensation by the lower limbs was not evaluated. We did not employ simultaneous acquisition of biplanar whole-body projections; resources such as the EOS imaging system (Biospace, Paris, France) can provide a complete picture of the spinal deformity and reveal any compensatory mechanisms engaged, with a significantly lower radiation dose than a single lumbar spine view. However, such resources are rarely available and no such system is currently available at our institution. Furthermore, there are notable sex-related differences in the risk factors for osteoporotic vertebral fracture^**(^30^)**^. Therefore, further studies are needed in order to determine whether the current findings can be applied to male patients with osteoporosis.

## CONCLUSION

It seems that patients with osteoporosis who develop fractures have worse global sagittal alignment, as evidenced by the spinopelvic parameter GT, than do those who do not develop such fractures. Higher GT values appear to correlate with an increased risk of fractures and with greater fracture severity, as measured by the SDI.

## Data Availability

The data generated or analyzed in the preparation of this study are included in this published article.

## References

[1] Jackson SA, Tenenhouse A, Robertson L. (2000). Vertebral fracture definition from population-based data:
preliminary results from the Canadian Multicenter Osteoporosis Study
(CaMos). Osteoporos Int.

[2] Bliuc D, Nguyen ND, Milch VE (2009). Mortality risk associated with low-trauma osteoporotic fracture
and subsequent fracture in men and women. JAMA.

[3] Pluijm SM, Tromp AM, Smit JH (2000). Consequences of vertebral deformities in older men and
women. J Bone Miner Res.

[4] Schousboe JT. (2016). Epidemiology of vertebral fractures. J Clin Densitom.

[5] Vokes TJ, Giger ML, Chinander MR (2006). Radiographic texture analysis of densitometer-generated calcaneus
images differentiates postmenopausal women with and without
fractures. Osteoporos Int.

[6] Kaneko A, Naito K, Nagura N (2020). Characteristics of sagittal spine alignment in female patients
with distal radius fractures due to fall. Heliyon.

[7] Cangussu-Oliveira LM, Porto JM, Freire RC (2020). Association between the trunk muscle function performance and the
presence of vertebral fracture in older women with low bone
mass. Aging Clin Exp Res.

[8] Peres-Ueno MJ, Capato LL, Porto JM (2023). Association between vertebral fragility fractures, muscle
strength and physical performance: a cross-sectional study. Ann Phys Rehabil Med.

[9] Lee JS, Lee HS, Shin JK (2013). Prediction of sagittal balance in patients with osteoporosis
using spinopelvic parameters. Eur Spine J.

[10] Zhang YL, Shi LT, Tang PF (2017). Correlation analysis of osteoporotic vertebral compression
fractures and spinal sagittal imbalance. Orthopade.

[11] Yokoyama K, Kawanishi M, Yamada M (2017). Age-related variations in global spinal alignment and sagittal
balance in asymptomatic Japanese adults. Neurol Res.

[12] Ishikawa Y, Miyakoshi N, Kasukawa Y (2013). Spinal sagittal contour affecting falls: cut-off value of the
lumbar spine for falls. Gait Posture.

[13] Dai J, Yu X, Huang S (2015). Relationship between sagittal spinal alignment and the incidence
of vertebral fracture in menopausal women with osteoporosis: a multicenter
longitudinal follow-up study. Eur Spine J.

[14] Marks M, Stanford C, Newton P. (2009). Which lateral radiographic positioning technique provides the
most reliable and functional representation of a patient’s sagittal
balance?. Spine (Phila Pa 1976).

[15] Savarese LG, Menezes-Reis R, Bonugli GP (2020). Spinopelvic sagittal balance: what does the radiologist need to
know?. Radiol Bras.

[16] Genant HK, Wu CY, van Kuijk C (1993). Vertebral fracture assessment using a semiquantitative
technique. J Bone Miner Res.

[17] Kerkeni S, Kolta S, Fechtenbaum J (2009). Spinal deformity index (SDI) is a good predictor of incident
vertebral fractures. Osteoporos Int.

[18] Matsunaga T, Miyagi M, Nakazawa T (2021). Prevalence and characteristics of spinal sagittal malalignment in
patients with osteoporosis. J Clin Med.

[19] Miyakoshi N, Kudo D, Hongo M (2017). Comparison of spinal alignment, muscular strength, and quality of
life between women with postmenopausal osteoporosis and healthy
volunteers. Osteoporos Int.

[20] Chau LTC, Hu Z, Ko KSY (2021). Global sagittal alignment of the spine, pelvis, lower limb after
vertebral compression fracture and its effect on quality of
life. BMC Musculoskelet Disord.

[21] Asahi R, Nakamura Y, Kanai M (2022). Association with sagittal alignment and osteoporosis-related
fractures in outpatient women with osteoporosis. Osteoporos Int.

[22] Ohnishi T, Iwata A, Kanayama M (2018). Impact of spino-pelvic and global spinal alignment on the risk of
osteoporotic vertebral collapse. Spine Surg Relat Res.

[23] In T, Lu J, Zhang Y (2021). Does spinal sagittal imbalance lead to future vertebral
compression fractures in osteoporosis patients?. Spine J.

[24] Fechtenbaum J, Etcheto A, Kolta S (2016). Sagittal balance of the spine in patients with osteoporotic
vertebral fractures. Osteoporos Int.

[25] Savarese LG, Menezes-Reis R, Jorge M (2022). Sagittal balance and intervertebral disc composition in patients
with low back pain. Braz J Med Biol Res.

[26] Obeid I, Boissière L, Yilgor C (2016). Global tilt: a single parameter incorporating spinal and pelvic
sagittal parameters and least affected by patient
positioning. Eur Spine J.

[27] Banno T, Togawa D, Arima H (2016). The cohort study for the determination of reference values for
spinopelvic parameters (T1 pelvic angle and global tilt) in elderly
volunteers. Eur Spine J.

[28] Yokoyama K, Ikeda N, Tanaka H (2024). Changes in spinal sagittal balance after a new osteoporotic
vertebral compression fracture. Osteoporos Int.

[29] Hu Z, Man GCW, Kwok AKL (2018). Global sagittal alignment in elderly patients with osteoporosis
and its relationship with severity of vertebral fracture and quality of
life. Arch Osteoporos.

[30] Roy DK, O’Neill TW, Finn JD (2003). Determinants of incident vertebral fracture in men and women:
results from the European Prospective Osteoporosis Study
(EPOS). Osteoporos Int.

